# Extraction, Purification, Bioactivities and Application of Matrix Proteins From Pearl Powder and Nacre Powder: A Review

**DOI:** 10.3389/fbioe.2021.649665

**Published:** 2021-04-20

**Authors:** Jingying Pei, Yan Wang, Xianguo Zou, Huajun Ruan, Changming Tang, Jie Liao, Guangjie Si, Peilong Sun

**Affiliations:** ^1^College of Food Science and Technology, Zhejiang University of Technology, Hangzhou, China; ^2^Key Laboratory of Food Macromolecular Resources Processing Technology Research, China National Light Industry, Hangzhou, China; ^3^Zhejiang Fenix Health Science and Technology Co., Ltd., Zhuji, China

**Keywords:** pearl powder, nacre powder, extraction, purification, bioactivities, application

## Abstract

Natural pearls are formed when sand or parasites (irritants) accidentally enter into the oyster body and form pearls under the cover of the nacre layer. Pearl powder is a powdery substance by grinding pearls into small grains, however, the nacre powder is the inner layer of outer corner layer and middle prism layer. Pearl medicine in China has a history of more than 2,000 years, pearl has the effects of calming the mind, clearing the eyes, detoxifying the muscle and so on. In this paper, the researches on the extraction of pearl powder and nacre powder, the isolation and purification of matrix protein and the various biological activities (osteogenic activity, antioxidant, anti-inflammatory, anti-apoptotic, promoting the migration of fibroblasts, and so on) are reviewed in detail. To provide readers with a faster understanding, the method of extraction and purification and the application of nacre powder and pearl powder are clearly presented in the form of figures and tables. In line with the concept of waste or by-product, there are more reports of nacre extract than pearl extract, due to the expensive and limited in origin of pearls. Mainly on the direct use of nacre powder and pearl powder or on the use of extracts (mainly water soluble proteins) through experiments *in vivo* or *in vitro*, and shows whether it is effective through the results of various indexes. There is no further study on substances other than extracts, and the structural analysis of extracts needs further exploration.

## Introduction

Pearl is an ancient organic gem and produced only from shellfish mollusks, which is a typical biomineralization material. Natural pearls are formed when sand or parasites (irritants) accidentally enter into the oyster body and form pearls under the cover of the nacre (Jin et al., 2019). At present, most of the pearls in the market are made by human intervention, rich in variety, with different shapes and colorful. Mother-of pearl commonly adopted included *Pinctada maxima*, *Pteria penguin*, *Pinctada martensii*, *Pinctada fucata*, *Pinctada margaritifera*, etc.

Pearl powder is a kind of powdery substance formed by crushing and grinding pearls, and can be divided into two categories: freshwater pearl powder and sea water pearl powder. The nacre is a substance secreted by the pearls sac and accumulated alternately by many crystalline layers of calcium carbonate and chitosan protein. Nacre powder is made from the inner part of the pearl oyster shell (State Pharmacopoeia Commission, 2020). The nacre powder is from the pearl oyster shell inner wall which removes the outer cuticle of the shell and the inner layer of the middle prismatic layer (pure pieces of nacre cutted from pearl oyster shells), the structure formed by calcium carbonate and conchiolin is called nacre. Nacre powder can also be divided into fresh water nacre powder and sea water nacre powder. Shell powder refers to the powder made by crushing and grinding shells, is different from nacre powder.

Pearls are secreted by the epithelium of the pearl bag whereas the shell nacre is secreted by the external epithelium of mantle, and their components are consistent. Nacre and pearl contain 95% calcium carbonate and about 5% organic matter, and also include a variety of amino acids, a variety of trace elements, rich vitamins and peptides. The biological activity components in pearl and nacre were organic matrix, which was a mixture of protein, polypeptide, glycoprotein, chitin, lipid, and pigment (Huang et al., 2010; He et al., 2016).

Pearl powder has the effects of calming the mind, soothing the liver and protecting the liver, regulating endocrine, clearing heat and detoxifying, and can supplement trace elements and promote metabolism. It can clear heat and detoxify, improve sleep, the symptoms of constipation and disease resistance, regulate endocrine and enhance the immunity of the body. Pearl powder is a beneficial source of calcium supplement (Gao et al., 2008). Pearl powder is often used for traditional Chinese medicine beauty and the treatment of various diseases, and it can promote the regeneration of skin collagen cells, enhance the elasticity of the skin, remove acne and delay aging (Yang et al., 2016, 2018a). Pearl powder is weakly alkaline, long consumption will adjust the pH value of blood, making blood weakly alkaline to prevent mosquito bites, improve skin immune function, strengthen stomach by neutralizing stomach acid, and exhibit anti-allergic effect. In recent years, pearl powder has made progress in the treatment of antioxidant system damage and hemolytic disease. In addition, pearl powder was found to enhance the adhesion of skin fibroblasts and tissue regeneration, playing an irreplaceable role in wound healing (Li, 2010). In terms of weight loss and body shaping in women, pearl powder can reduce weight, visceral fat, and blood triglyceride levels. Pearl powder is a valuable traditional Chinese medicine for the treatment of palpitation, convulsion or epilepsy for thousands of years, but its active ingredient is still unknown (Wu, 2001; Zhang W. D. et al., 2003; Yang et al., 2012, 2018b).

The nacre powder has the function of soothing the mind, clearing heat and detoxifying, and was used for neurasthenia, pharyngitis, external treatment of tongue swelling and pain (Zhang et al., 2008; Duan and Dong, 2009). Nacre powder has excellent features such as availability, mechanical resistance, biocompatibility, biodegradability, X-ray opacity and osteogenic properties (Shen et al., 2006; Kim et al., 2012; Brion et al., 2015; Gerhard et al., 2017). Implantation into the bone environment can release signaling molecules and induce osteogenesis. Water-soluble matrix (WSM) in nacre powder plays a major role in controlling the formation of bone interface structure, biocompatibility with bone, and stability of biological tissue (Mao and Xu, 2016).

Scientific studies have shown that the composition and content between nacre powder and pearl powder is very close. However, in the process of nacre powder, a small amount of alkaline residue was formed, which will cause certain stimulation to the skin, leading to damaged skin acid-base balance after long-term consumption of products with high pH (Zhang W. D. et al., 2003). Compared with nacre powder, pearl powder has higher price and consumer recognition, and more products in China. Pearl powder is often used in skin whitening, freckle and acne removal, but it is less used in practical production in other fields (health products, pharmaceuticals, material applications, textiles). Pearl powder has good applications in improving eyesight, eliminating halo, preventing leakage and so on. It is a new research hotspot in osteogenesis, weight loss and calcium supplementation. Pearl powder is used in the treatment of oral ulcer, gastric ulcer, duodenal ulcer, palpitation, spasm or epilepsy and other diseases. Nacre powder is seldom used in cosmetics due to its complex processing technology and low product quality. In order to improve the utilization rate of pearls and expand the application field, the key issues to be solved in pearl industry are to understand the biological activity and mechanism of pearl powder and nacre powder (Xia et al., 2020).

Recently, freshwater pearls (*Hyriopsis cumingii Lea*) have gradually replaced seawater pearls. The yield of sea pearl is low, which produces 1–3 pearls per individual. The yield of fresh water pearls is relatively high, averaging 6–8 pearls per shellfish. Freshwater pearl culture cycle is short and the pearl breeders can make pearls big, round and colorful. Freshwater pearl can be comparable to those of seawater pearls, which have little difference in composition with seawater pearls.

In this paper, the extraction (preparation and separation) of pearl powder and nacre powder, the isolation and purification of matrix protein, the bioactivity (osteogenic activity, antioxidant, anti-inflammatory, anti-apoptotic, promoting the migration of fibroblasts and so on) and their application fields are described in detail and systematically. A comprehensive review of the extraction method, purification method and application range of nacre powder and pearl powder is provided. The research and utilization of nacre are more reported than pearl, which is waste utilization. The cost of pearl is higher, and there are less reports on pearl extract than nacre extract. The author mainly does some experiments *in vivo* or *in vitro*, and shows whether it is effective through the results of various indexes. There is no further study on substances other than extracts, and the structural analysis of extracts needs further exploration.

## Extraction and Purification

The composition and content of pearl and nacre are very similar as shown in Table 1 (Pu et al., 2016). A comparison and summary of the trace elements, amino acid content, preparation method and matrix protein separation and purification of the two were listed as follows.

**TABLE 1:** The types and contents of trace elements in different kinds of pearl powder and nacre powder.

	**Name**	**Trace elements (mg/kg)**										
		**Na**	**Co**	**As**	**Sr**	**Mn**	**Ba**	**Fe**	**Mg**	**K**	**B**	**Cr**
1	*Hyriopsis cumingii* shell nacre	1028.0∼2566.0	0.7∼1.0	7.8∼24.2	93.0∼207.0	15.7∼117.5	15.0∼67.5	2.1∼60.2	0.9∼1.0	0.7∼8.7	0∼1.1	/
2	*Hyriopsis cumingii* pearl	1012.0∼2065.0	0.8∼0.9	12.2∼17.5	108.0∼159.0	24.6∼138.4	23.4∼61.0	3.7∼4.1	0.6∼3.4	0∼2.5	/	0.6∼3.9
3	*Hyriopsis cumingii* natural pearl	2012.0	0.9	15.5	119.0	18.1	26.7	/	0.7	/	/	0.6
4	*Pinctada martensii* shell nacre	4573.0∼4939.0	0.7∼0.9	9.7∼15.8	306.0∼351.0	0∼0.6	/	0∼1.5	4.0∼6.5	7.5∼28.4	1.0∼2.7	/
5	*Pinctada martensii* natural pearl	5462.0	0.7	14.3	261.0	/	/	/	9.0	15.2	0.2	/
6	*Pinctada maxima* (gold-lip) shell nacre	5057.0	0.7	13.1	285.0	/	/	3.0	2.7	16.9	4.4	/
7	*Pinctada margaritifera* (black lip shell) shell nacre	5191.0	0.7	12.0	300.0	1.7	/	0.6	4.2	20.0	2.8	
8	*Pteria penguin* shell nacre	4561.0	0.7	15.7	349.0	/	/	/	3.9	10.2	2.4	
9	Sea pearl	5741.0	/	16.2	260.0	/	23.3	1.0	4.0	8.7	1.7	

### Trace Elements and Amino Acids

There are slight differences in chemical composition among pearls with different colors (Jiang, 2019). In shell nacre and pearls, the content of Sr, Na elements in pearl is higher, while the content of Mg, Fe elements in natural pearl is lower. There is no obvious difference in the element composition between pearl and nacre in seawater.

Most freshwater pearls contain 20 amino acids, including 11 non-essential amino acids and 7 essential amino acids. The species and total amount of amino acid in freshwater pearl are more than that of in nacre. There are 22 kinds of amino acids in seawater pearl, and the content of them is obviously higher than that of the freshwater pearl, among which five kinds are the newly discovered non-proteolytic amino acids, and the content of taurine is the highest (Zheng and Mao, 2004; Wang, 2016).

### Preparation of Pearl Powder and Nacre Powder

Pearl powder can be divided into micron pearl powder and nano-pearl powder (NPP) according to particle size. The main processing method is grinding. The preparation of NPP is to employ the stirring ball mill to grind and refine the micron-sized pearl powder. The particle size of pearl powder is detected by Malvern laser particle size analyzer, transmission electron microscope (TEM) and scanning electron microscope (SEM).

The preparation methods of nacre powder include mechanical crushing, acid hydrolysis and alkali hydrolysis.

Mechanical crushing: Grinding off the cuticle and prismatic layer of the shell and remaining nacre. After grinding and sieving, nacre powder is obtained (Li, 2001).

Acid hydrolysis: Nacre powder with 5% dilute hydrochloric acid (HCl) and sterilize with 0.5% hydrogen peroxide and then freeze-dried. This method corrodes equipment and pollutes the environment, which is not suitable for mass production (Zhang J. G. et al., 2003).

Alkali hydrolysis: Soak shells with sodium hydroxide (NaOH), exfoliate cuticle and prismatic layer. However, there was NaOH residue in the samples. Products can damage the balance of the skin acid and base. So the nacre after alkali treatment should be washed with water for many times or treated with acid to reduce the residue of alkali (Sun, 2011).

### Isolation and Purification of Matrix Protein

#### Isolation and Purification of Pearl Powder Matrix Protein

The pearl powder was dissolved or enzymolyzed to obtain the pearl powder extract, the supernatant was centrifuged to obtain the mixed solution of various amino acids, trace elements and peptides, and then the mixed solution was separated and purified. The extract of fresh water pearls powder were divided into WSM, water-insoluble matrix (WISM), acid-soluble protein (ASP), and the more studied were WSM (Ma et al., 2011). WSM is highly water-soluble, and can be widely used in cell experiments and skin wound healing experiments. Its active components can be absorbed by cells and skin quickly, while the insoluble proteins are granular or lumps insoluble in water, which is not conducive to the conduct of various experiments.

The preparation methods of pearl powder extract include acid-enzymatic hydrolysis, enzymatic hydrolysis, acid hydrolysis, ultrasonic hydrolysis, supercritical CO_2_ extraction, ammonium sulfate-precipitation and so on. Microwave method and ultrasonic method are time-consuming, and the protein extraction rate of enzymatic and acid hydrolysis method needs to be further improved. The widely used preparation method is enzyme-acid hydrolysis method, which has high protein yield, and is simple processing and easy operation. The following listed are some common preparation methods (Table 2).

**TABLE 2:** Summary of the extraction and purification processes of pearl powder matrix protein.

**Methods**	**Extraction solvent**	**Principles**	**Reaction conditions**	**Advantages and disadvantages**	**References**
Water extraction	deionized water	The extracts with molecular weight greater than 5 kDa and less than 5 kDa were separated by centrifugal membrane concentration filter device and ammonium sulfate precipitation method, and then the soluble protein and insoluble protein were separated.	Room temperature, 1∼3 h	The operation was simple, the reaction conditions were mild, and the protein with different molecular weight and water solubility were obtained simultaneously.	Bedouet et al. (2001)
Acid extraction	weak acid	The precipitation was obtained by centrifugation at ultra-high pressure and low temperature, and the water-soluble protein and acid-soluble protein were obtained by using the difference between the water-soluble protein and acid-soluble protein	30∼50°C, 0.5∼3 h	Water soluble pearl protein and acid soluble pearl protein were obtained by simple operation and mild reaction conditions.	Zhang J. G. et al. (2003), Jin et al. (2019)
Enzyme-acid extraction	phosphate buffer (pH 6.5∼7.5)	Phosphoric acid buffer was added to extract acid-soluble protein, and then neutral protease was added to hydrolyze the active peptide.	Add phosphate buffer, 100∼120°C, 0.15∼0.2 Mpa, 20∼60 min, after adding the neutral protease, 40∼70°C, 4∼8 h	High reaction temperature, long time, protein inactivation.	Kim et al. (2012); Kishore and Southgate (2015)
Supercritical CO_2_ extraction	deionized water (pH 3.0)	Supercritical CO_2_ extraction method and acetic acid/sodium acetate buffer were used.	25∼60°C, 10∼80 Mpa, 0.5∼10 h	Safe, non-toxic, cheap, strong extraction ability, high extraction rate; Fast extraction time and short production cycle	Tsukamoto et al. (2004); Lao et al. (2007)

#### Isolation and Purification of Nacre Powder Matrix Protein

The methods of nacre powder extract is not mature, usually using water extraction and alkali extraction (Table 3). Buffers used in alkali extraction include ethylene diamine tetraacetic acid tetrasodium (EDTA-4Na pH 7.6), ammonium hydroxide (NH_4_OH pH 8.5), disodium hydrogen phosphate-potassium phosphate monobasic (Na_2_HPO_4_-KH_2_PO_4_) and NaOH.

**TABLE 3:** Summary of the extraction and purification processes of nacre powder matrix protein.

**Methods**	**Extraction solvent**	**Principles**	**Reaction conditions**	**Advantages and disadvantages**	**References**
Water extraction	Deionized water	Continuous stirring, centrifugation, filtration supernatant, freeze-drying.	RT, 1∼3 h	The operation was simple, the reaction conditions were mild.	Bédouet et al. (2007)
Alkali extraction	EDTA-4Na (pH 7.6), NH_4_OH (pH 8.5)	Dialysis and separation yielded WSM and WISM, extracted WISM with NH_4_OH, ALSM (acid water-soluble matrix) was obtained by centrifugation.	RT	WSM, WISM and ALSM can be separated simultaneously	Samata et al. (1999)
	Na_2_HPO_4_-KH_2_PO_4_	Freeze drying, dissolution and dialysis of concentrate.	RT	The operation was simple, fast extraction time	Zhang et al. (2016)
	NaOH	Grinding powder, dialysis with acetic acid. After complete decalcification, soluble organic matrix and insoluble organic matrix were separated.	RT	The operation was simple, economy is applicable, soluble organic matrix and insoluble organic matrix were separated simultaneously	Zhang et al. (2006)

*RT, room temperature.*

Samples obtained by the above experimental methods were analyzed by high performance liquid chromatography (HPLC), sodium dodecyl sulfate polyacrylamide gel electrophoresis (SDS-PAGE), matrix-assisted laser-desorption ionization-time-of-flight mass spectrometer mass spectrometry (MALDI-TOF MS) and *N*-terminal sequencing (Zhang et al., 2015). To date, more than 50 proteins and more than 50 peptides have been identified from the nacre (UniProt protein database, www.uniprot.org).

There are many technical methods to identify true and false pearl powder (nacre powder, shell powder) through whether or not have prismatic crystalline layers, for example, X-ray diffraction (XRD) identification method, using the XRD diagram of the sample to be tested for phase determination. Spectral imaging analysis technology can be used to construct the fingerprint of pearl powder, and can quickly and accurately identify different pearl products and their authenticity. Compared with the traditional method, it is more simple, convenient, effective and stable in quality evaluation and quality identification. The above are the differences of several detection methods, but it is necessary to establish a unified standard detection method. Therefore, two national standards, “pearl powder (GB/T 36930-2018)” and “X-ray diffraction analysis (GB/T 36923-2018)” for pearl powder identification methods, have been officially released and implemented in China on July 1, 2019. Using X-ray diffractometer, some scholars observed the phase change rate of pearl powder and shell powder (a crystal, made by crushing and grinding shells) under condition of 380∼400°C (Zhang et al., 2013).

## Biological Activities

Nacre Powder and extraction, pearl powder and extraction have a variety of biological activities as shown in Table 4.

**TABLE 4:** Summary of the effect of nacre powder and extraction, pearl powder and extraction.

**Biological Activity**	**Material**	**Freshwater or seawater**	**Principles**	***in vivo* or *in vitro***	**References**
Osteogenic Activity	Nacre powder	Freshwater and seawater	It had proliferation effect on osteoblasts, osteoclasts and bone marrow cells, which can treat loss of vertebral bone.	*in vivo* and *in vitro*	Liao et al. (1997); Almeida et al. (2000), Lamghari et al. (2001); Rousseau et al. (2003), Asvanund et al. (2011); Asvanund and Chunhabundit (2012), Mao and Xu (2016); Zhang et al. (2017)
	Pearl powder	freshwater	promoted cartilage regeneration (vertebral bone in rats, sheep knee joint)	*in vivo*	Berland et al. (2005)
	Pearl powder extract	/	Had proliferation effect on osteoblasts	*in vitro*	Kim et al. (2012)
Antioxidant Activity	Pearl powder	freshwater	It increased total antioxidant capacity, total thiols (SH group) and glutathione content with suppressing lipid peroxides products.	*in vivo* and *in vitro*	Chiu et al. (2018)
		/	It significantly inhibited membrane lipid peroxidation and protein oxidation.	*in vitro*	Yang et al. (2017)
	Nacre powder extraction (WSM)	/	It had free radical scavenging ability and inhibition of lipid peroxidation	*in vitro*	Chaturvedi et al. (2013)
Promoting Fibroblast Migration	Nacre powder	Freshwater	Nacre powder also promoted extracellular matrix synthesis and intercellular adhesion attachment and tissue regeneration (type I and type III collagen).	*in vitro*	Lopez et al. (2000); Chen et al. (2019)
	Pearl powder extraction (WSM)	Freshwater	WSM and MR14 can significantly promote the proliferation of fibroblasts and the accumulation of collagen.	*in vitro*	Dai et al. (2010)
	Pearl powder extraction	Freshwater	Promote wound healing	*in vitro*	Li et al. (2013)
	Nacre powder extraction (WSM)	Sseawater	WSN improved the healing process of burn wounds by rapidly restoring angiogenesis and fibroblast activity.	*in vivo*	Li (2010)
Anti-inflammatory and Anti-apoptotic Effects	Pearl powder extraction	/	Inhibited radiation dermatitis occurring in keratinized cells	*in vitro*	Yang et al. (2015)
Other Activities	Pearl powder	freshwater	Pearl powder was a beneficial calcium source for adults, nanocrystallization improved calcium bioavailability of pearl powder.	*in vivo*	Chen et al. (2008); Shono et al. (2008), Dai et al. (2010)
Bone Repair Material	Pearl/Polypropylene	freshwater	Pearl powder enhanced the biocompatibility of the monofilament, which could enhance the cell activity of the polypropylene monofiber.	*in vitro*	Deng et al. (2015)
	Pearl/PLGA	freshwater	Compared with tricalcium phosphate (TCP)/PLGA scaffolds, pearl/PLGA scaffolds has better biocompatibility and bone conductibility.	*in vitro*	Li et al. (2020)
	Pearl/PLLA Nacre/PLLA	freshwater	Pearl powder/PLLA and nacre powder/PLLA composite scaffolds could promote mouse bone marrow mesenchymal stem cell proliferation and increase ALP activity.	*in vitro*	Liu et al. (2014)
	Pearl/PAA	freshwater	Cells grown on the composite surface showed higher ALP activity, more calcium nodule formation.	*in vitro*	Wu et al. (2019)
New Technology	Pearl fiber	freshwater	The material is not only soft and comfortable, moisture absorption and breathable, but also easy to dye, antistatic.	*in vitro*	Liao (2018); Hu and Wu (2020), Liu et al. (2020)

*“/” indicated that no specific explanation in this reference.*

### Osteogenic Activity

Compared with autologous bone graft, hydroxyapatite coated titania (THA), β-tricalcium phosphate (β-TCP) and polymethylmethacrylate (PMMA), nacre was well tolerated by the host tissue and stimulated a faster osteogenesis (Lamghari et al., 2001; Asvanund et al., 2011; Asvanund and Chunhabundit, 2012; Zhang et al., 2017).

Nacre powder has better biodegradability and biocompatibility, and its osteogenic effect is more prominent. Nacre powder has a strong proliferation effect on osteoblasts and osteoclasts in the process of bone tissue formation and morphogenesis (Mao and Xu, 2016).

Compared with β-TCP microparticles, nacre powder (*Pinctada maxima*) can better promote osteoblast synthesis of sialoprotein in jaw osteoblasts and induce the express ion of bone-specific markers alkaline phosphatase (ALP) genes (Liao et al., 1997).

To further confirm the osteogenic activity of nacre powder, Lamghari et al. (1999) conducted *in vivo* and *in vitro* studies of nacre powder (*Pinctada margaritifera*) through stimulating bone marrow cells and bone formation to test whether nacre powder was suitable for the treatment of vertebral bone loss. *In vitro* study of bone marrow cells in rats by using nacre powder extract found that the stimulating effect of the extract on bone marrow cells was enhanced, and the ALP activity was also enhanced. The reduction in cell proliferation after WSM treatment is similar to that reported when cultured bone cells incubated tumor growth factor-b (TGF-β) or BMP, which are members of the TGF-β superfamily. The reduction in cell number after incubation with WSM was followed by a marked increase in ALP activity, an effect very like that of TGF-β and BMPs. This sequence of changes suggested that WSM has a selective action on potentially ostegenic cells within the bone marrow population. Thus, both the *in vivo* and *in vitro* findings suggested that nacre contains one or more signal molecules capable of activating osteogenic bone marrow cells (Almeida et al., 2000; Rousseau et al., 2003).

Pearl powder not only plays an important role in the recovery of vertebral bone in rats, but also has a good effect on cartilage regeneration. To investigate the role of pearl powder in the subchondral bone implantation of sheep knee joint and the role in the synovial membrane of femur in the subchondral area of sheep knee joint. The results showed that the implant had good tolerance without any synovial inflammation and regenerated new cartilage was observed after 3 months (Berland et al., 2005).

Not only pearl powder and nacre powder have osteogenic activity, but also pearl powder extract has the ability to promote the proliferation and differentiation of osteoblasts. The extract of nacre powder (*Pinctada maxima*) has the factor of inducing signal transduction, which could increase the amount of lymphocyte tumor-2 gene (B-cell lymphpma-2, Bcl-2) in B cytoplasm and nucleus (which can inhibit apoptosis), promote osteoblast proliferation and differentiation and induce osteogenesis (Kim et al., 2012).

Moreover, Duplat et al. (2007) cultured rabbit osteoclasts in culture medium containing WSM (*Pinctada margaritifera*) and cortical bone slices. The results showed that the inhibitory effect of papain, cathepsin K, B, L and the degree of bone slice absorption were all affected by WSM, but the inhibition of adhesion and proliferation of osteoclasts was not obvious. It can be preliminarily concluded that the activity of cysteine protease may be specifically inhibited by some organic molecules in the nacre, resulting in the decrease of bone resorption activity of osteoclasts. The nacre may contain certain bone remodeling factors that activate osteoblasts and regulate c-jun amino-terminal kinase (JNK) and Fos-related antigen-1 (Fra-1) protein signaling transduction to promote osteoblast mineralization (Duplat et al., 2007).

### Antioxidant Activity

In order to further explore the effects of pearl powder on the antioxidant activity of human body *in vitro* and *in vivo*. During *in vitro* studies, Chiu et al. (2018) evaluated various oxidation indices, along with lifespan-prolonging effect were checked using wild-type *Caenorhabditis elegans*. The clinical trial recruited 20 healthy middle-aged subjects, divided into experimental group and placebo group, given pearl powder 3 g/d and placebo 3 g/d for 8 weeks (Chiu et al., 2018). As a result, the abundant presence of protein content (amino acids and minerals) in pearl powder demonstrated an increased total antioxidant capacity, total thiols (SH group) and glutathione content with suppressing lipid peroxides products. It was proved that pearl powder can be used to treat various age-related degenerative diseases.

In addition, pearl powder also has a strong anti-hemolysis effect *in vitro*. Yang et al. (2017) investigated the *in vitro* antihemolytic and antioxidant properties of pearl powder that could protect erythrocytes against 2,20-azobis (2-amidinopropane) dihydrochloride (AAPH)-induced oxidative damage to membrane proteins and lipids. Pearl powder significantly inhibited membrane lipid peroxidation and protein oxidation, and increased the activity of superoxide dismutase (SOD) and glutathione peroxidase (GSH) levels (Yang et al., 2017).

While Chaturvedi et al. (2013) cultured MC3T3-E1 cells (a clonal preosteoblastic cell line originated from new-born mouse calvaria) and human epidermal cells (HaCaT cells) using medium containing WSM (nacre). Compared with the control group, the activity of intracellular ALP was significantly increased, osteocalcin (OCN) and collagen-1 A2 were significantly increased, the bone mineralized nodules were formed obviously, and had significant *in vitro* ABTS free radical scavenging ability, DPPH free radical scavenging ability and inhibition of lipid peroxidation (Chaturvedi et al., 2013).

### Promoting Fibroblast Migration

Water-soluble matrix and WISM from pearl powder was extracted, and the insoluble residue was demineralized, size-fractionated, and named as MR14 (>14 kDa), MR3-14 (3–14 kDa), MR3 (<3 kDa). WSM and MR14 can significantly promote the proliferation of fibroblasts and the accumulation of collagen, and each component can significantly promote the production of metalloproteinase-1 (timp-1), which MR14 can significantly inhibit the activity of matrix metalloproteinase-2,-9 (mmp-2) (Dai et al., 2010).

Moreover, the composition of nacre powder extract also has the effects of promoting the healing and recovery of burn wounds. Water soluble nacre (WSN) and WSM is responsible for promoting healing and recovery of burn wounds. The main ingredients are water-soluble proteins, a small amount of vitamins and trace elements. The nacre (*Pteria Martensii*) plays an induction role in deep secondary burn wound healing in pigs. When WSN was applied to burn areas, granulation tissue was rapidly filled with collagen, and the damaged dermis and epidermis returned to the appearance of normal skin, indicating that WSN improved the healing process of burn wounds by rapidly restoring angiogenesis and fibroblast activity (Dai et al., 2010). WSN and WSM both promote the expression of collagen gene and achieves wound repair.

Pearl powder extract has the effects of promoting cell migration, which can be used as a supplement to cell culture and help to promote wound healing (Li et al., 2013). The extract promotes the expression of I collagen gene and achieves wound repair. Nacre (*Pinctada maxima*) also promotes extracellular matrix synthesis and intercellular adhesion attachment and tissue regeneration (type I and type III collagen) (Lopez et al., 2000; Chen et al., 2019).

### Anti-Inflammatory and Anti-Apoptotic Effects

As a result of acute radiation-induced skin reactions and injuries, radiation therapy often has side effects on cancer patients. Clinical manifestations are skin irritation, pruritus, desquamate, pigmentation, ulcer bleeding and other symptoms. In addition to causing discomfort and affecting the quality of life, it can also increase the risk of local or systemic infection and lead to interruption of radiotherapy. At present, there is no uniform treatment for acute radiation dermatitis, and the evaluation of various methods is not consistent. To improve this phenomenon, the main active components of pearl (WSMs, high purity natural amino acid) and its anti-inflammatory and anti-apoptotic effect were studied (Yang et al., 2015). With different concentrations of the bead extract, the effects of low-dose outdoor ultraviolet (UVB) on immortalized HaCaT cells were investigated. And the results showed that pearl powder extract was non-toxic to HaCaT cells and had the potential to inhibit radiation dermatitis occurring in keratinized cells.

The nacre powder extract was test on artificially dehydrated skin explants and the expression of polyfilament protein and transglutaminase 1 in dehydrated skin after labeling with specific monoclonal antibodies was observed. This study found that nacre powder extract can induce the reconstruction of intercellular cements in cuticle and can be used to treat dermatitis symptoms.

### Other Activities

Pearl powder is a beneficial source of calcium supplement, and the particle size of pearl powder is an important factor affecting the utilization of calcium in human body. Both NPP and micro powder (MPP) prepared by dry powder grinding were studied (*Hyriopsis cumingii* lea) (Chen et al., 2008). The bioavailability was evaluated by the serum total calcium increment, the serum intact parathyroid hormone (iPTH) reduction, and the urine calcium/creatinine ratio increment in 6 h after administration. The results show better absorption and retention of calcium from NPP, as reflected with the shorter time elapsed before the maximum concentration of calcium appeared in the serum, higher iPTH reduction, more calcium absorption, and higher maximum calcium concentration (Cmax) in serum after ingestion, than that from MPP. Healthy adults with hyperthyroidism, hypercalcemia and hypocalcemia were selected as subjects for oral pearl powder. Bioavailability was evaluated by increasing serum total calcium increment, decreased serum parathyroid hormone (iPTH), and increased urinary calcium/creatinine ratio at 6 h after administration. The results showed that pearl powder was a beneficial calcium source for adults, nanocrystallization improved calcium bioavailability of pearl powder. At the same time, pearl powder also contained a variety of trace elements, which can supplement the minerals needed by the human body. Taking pearl powder (*Pinctada maxima*) could reduce body weight, visceral fat and blood triglyceride levels (Shono et al., 2008; Dai et al., 2010).

## Application of Pearl Powder

China is the world’s largest producer of freshwater pearls, accounting for about 95% of the world’s output. Seawater pearls in the world are mainly produced from Japan’s Akoya. Pearl processing centers are concentrated in China and Japan. In the 1990s, unscientific culture management and surgical practices in China led to shuddy production and an epidemic of mussel disease, which sent pearl prices to their lowest ebb. At present, the commodities in the pearl market are mainly pearl ornaments, and there is not enough research on the medicinal ingredients of raw materials. Therefore, the leading pearl enterprise is committed to the deep processing and technology research and development of pearl culture, to develop the market value of fresh water pearls.

### Traditional Application

Pearls are widely used in daily life, including pearl jewelry products, health products, personal care products, medicines, food additives, etc. Health care category, in the form of capsules and oral liquids to enter the market. Besides, there are whitening eye cream, facial mask personal care products and medications, including anti-hypertensive tablets and anti-inflammatory tablets. Pearl powder combined with other drugs can treat burn radiation dermatitis, hand, foot and mouth disease, oral ulcer and other diseases (Duan and Dong, 2009; Li, 2010).

### New Technology of Pearl Powder Application

In recent years, the application of pearl powder no longer stays in beauty, skin care, health products and other aspects, in composite support materials, fabric spinning has also made new progress.

In order to simulate the natural structure of bone, many researches on organic-inorganic composites have been carried out. Pearl has a special brick and mortar hierarchy, which is a good bone repair material with high osteogenic activity, but it is less used in clinical bone repair and reconstruction because of its brittle and difficult to form. The organic matrix in pearl powder has the characteristics of strong hydrophobicity and good interfacial equilibrium structure, which plays an important role in the determination of biocompatibility of biological shell tissue. When pearl powder is placed in a biological system, the organic matrix protein produces a new interface structure, which affects its interface inversion and should be compatible with biocompatibility. Furthermore, since surface charges strongly affect the surface reactivity of their biomaterials, the charge at the organic/inorganic interface should be considered as the dominant factor determining implant surface stability and regulating tissue properties.

### Pearl/Polypropylene

Deng et al. (2015) used pearl powder as raw material to prepare polypropylene (PP) monofilament with polypropylene resin, and its mechanical properties are similar to that of the original PP monofilament. The results showed that the presence of pearl powder enhanced the biocompatibility of the monofilament, which could enhance the cell activity of the polypropylene monofiber, promote cell proliferation, and then promote the biocompatibility of the polypropylene monofiber mesh (Deng et al., 2015).

### Pearl/PLGA

Xu et al. (2010) developed the pearl powder/poly (lactic-co-glycolic acid) (pearl/PLGA) porous composite scaffolds by low temperature deposition. Pearl/PLGA scaffold can promote the proliferation and increase the ALP activity of rabbit bone marrow stem cells (MSCs), and composite scaffolds showed a greater around the expression of type I collagen. Compared with TCP/PLGA scaffolds, pearl/PLGA scaffolds has better biocompatibility and bone conductibility (Li et al., 2020).

### Pearl/PLLA

Aragonite pearl powder, spherulite pearl powder and aragonite pearl powder (*Hyriopsis cumingii*) were combined with polylactic acid (poly-l-lactide, PLLA) to prepare three-dimensional porous composite. The results showed that both aragonite pearl powder/PLLA and nacre powder/PLLA composite scaffolds could promote mouse bone marrow mesenchymal stem cell proliferation and increase ALP activity. Among them, aragonite pearl powder/PLLA group was slightly higher than nacre powder/PLLA group, while aragonite pearl powder/PLLA composite scaffold cell proliferation and ALP activity decreased, which may be due to the increased solvent pH caused by partial dissolution of aragonite (Liu et al., 2014).

### Pearl/PAA

Wu et al. (2019) prepared pearl powder/polyamino acid (PAA) composites by *in situ* melt polycondensation to combine the high osteogenic activity of pearl with PAA flexibility. The compressive strength, flexural strength and tensile strength of composites reached 161 Mpa, 50 and 42 Mpa, cells grown on the composite surface showed higher ALP activity, more calcium nodule formation, and higher expression levels of genes associated with osteogenic differentiation than cells grown on the PAA surface.

### Pearl Fiber

**FIGURE 1:**
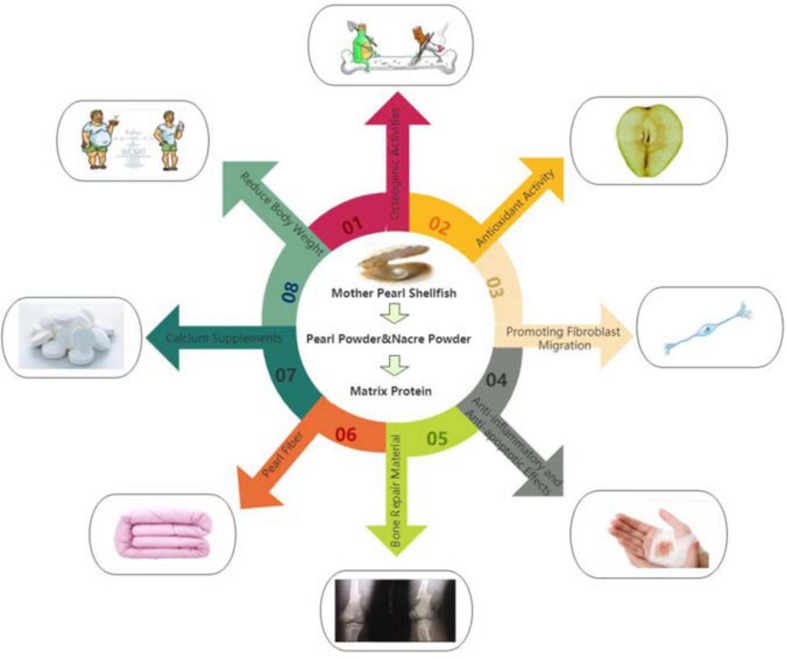
(01) Osteogenic activity of nacre powder and pearl powder. (02) Antioxidant activity of nacre powder and pearl powder. (03) The composition of nacre powder and pearl powder extract also has the effect of promoting the healing and recovery of burn wounds. (04) Anti-inflammatory and anti-apoptotic effects of nacre powder and pearl powder. (05) Pearl powder is used as bone repair materials. (06) Textile materials with pearl powder. (07) Calcium salts in pearl powder can be used as calcium supplements. (08) Proper administration of pearl powder can reduce fat.

Pearl fiber is the use of high-tech means of nanometer pearl powder in viscose fiber spinning into the fiber, so that the fiber body and appearance of the uniform distribution of nano-pearl particles. It is composed of pearl protein functional masterbatch and fiber grade resin section, which makes the nano pearl particles evenly distributed in the body and appearance of the fiber. Because the base material of pearl fiber is viscose fiber, the material is not only soft and comfortable, moisture absorption and breathable, but also easy to dye, antistatic. After the textile made, it is brought to the market, favored by consumers, and the products are in short supply. Pearl fiber can be mixed with a variety of materials to develop a comfortable and healthy fabric, such as cotton/viscose/pearl fiber, pearl fiber/combed cotton, etc (Hu and Wu, 2020; Liu et al., 2020; Liao, 2018). The biological activities of pearl powder and nacre powder as shown in Figure 1.

Based on the above conclusions, the researches on biological activity and action mechanism of pearl powder and nacre powder are mainly from cell experiment and animal experiment (rat, sheep, rabbit, and pig). However, further research is needed if it is to be applied in vertebrates or the human tissues or cells, which is also the direction of the development of pearl powder and nacre powder.

## Structure

The structures of matrix protein in nacre powder and pearl powder were summarized as shown in Table 5.

**TABLE 5:** Summary of the structure of nacre powder extraction.

**Mother oysters**	**Materials**	**Active ingredient**	**Molecular weight (kDa)**	**Principles**	**Single component (√ or/)**	**References**
unknown	nacre	WSM	0.05∼0.3	Reversed-phase HPLC (RP-HPLC), ESI	/	Rousseau et al. (2008)
*Pinctada fucata*	nacre	WSM (N16)	16	*N*-terminal amino acid determination, crystallization experiments,	√	Samata et al. (1999)
*Pinctada fucata*	nacre	Matrix Protein p10	10	HPLC, SDS-PAGE, MALDI-TOF, crystallization experiments, Raman microprobe spectroscopy, XRD	√	Zhang et al. (2006)
*Pinctada fucata*	nacre	WSM (P60)	60, 35, 28	SDS-PAGE, *N*-terminal sequencing, MALDI-TOF MS, amino acid analysis, crystallization	√	Lao et al. (2007)
*Pinctada maxima*	nacre	ALSM	9, 15, 11, 12, 20, 65	SDS-PAGE, amino acid analyses, *N*-terminal amino acid sequencing	/	Bedouet et al. (2001)
*Pinctada margaritifera*	nacre	WSM	0.5∼1	RP-HPLC	/	Duplat et al. (2007)
*Haliotis laevigata*	nacre	/	/	amino acid sequence, ESI, HPLC	/	Weiss et al. (2001)
*Pinctada margaritifera*	nacre	WSM	6∼8	RP-HPLC, SDS-PAGE, ESI	/	Bédouet et al. (2007)
*Hyriopsis cumingii*	pearl	WSM ASM	10, 25, 50, 10, 65	SDS-PAGE, XRD, Fourier transform infrared spectroscopy (FTIR)	/	Ma et al. (2011)

The studies mainly focused on the effect of nacre matrix proteins on the crystal morphology of calcium carbonate, which is divided into calcite, aragonite, and spheroid aragonite. At present, the single component of nacre extraction are P60, p10, and N16. The p10 plays important roles in controlling both calcium carbonate crystal formation. The most abundant amino acids in the p10 that account for 66.2% of the total residues are glycine (37.2%), alanine (13.3%), and lysine (15.7%) (Zhang et al., 2006). The P60 protein was a protein complex composed of several subunits with disulfide bridges. The known protein nacrein and its two derivatives, N28 and N35, were included in the P60 protein complex. The most abundant amino acids in the P60 that account for 68.3% of the total residues are glycine (32.1%), aspartic acid (17.4%), alanine (13.6%), and glutamic acid (5.2%). The *in vitro* study of the crystallization showed that this protein complex could control the formation and size of calcium carbonate (Lao et al., 2007). The study of single structure analysis of nacre matrix protein should be strengthened in the future.

## Conclusion

In this paper, the methods of preparation and separation of pearl powder and nacre powder, the processes of isolation and purification of matrix protein from pearl powder and nacre powder, the bioactivities (osteogenic activity, antioxidant, anti-inflammatory, anti-apoptotic, promoting the migration of fibroblasts, and so on) and their new technologies applied in pearl powder are described in detail and systematically. Pearl powder and nacre powder have no cytotoxicity, most studies reported *in vitro* pharmacological activities and *in vivo* studies from pearl powder and nacre powder, the mechanism of osteoblast *in vitro* and osteoblast *in vivo* of pearl powder and nacre powder needs further study. It is the hotspot to study the application of pearl power in safe, healthy and professional health care products and medicines in future.

## Author Contributions

JP: conceptualization and writing – original draft preparation. YW: conceptualization and writing, editing, and project administration. XZ: editing. CT, JL, and GS: validation. HR: project administration. PS: supervision and project administration. All authors contributed to the article and approved the submitted version.

## Conflict of Interest

HR, CT, JL, and GS was employed by the company Zhejiang Fenix Health Science and Technology. The remaining authors declare that the research was conducted in the absence of any commercial or financial relationships that could be construed as a potential conflict of interest.
